# Dataset on exogenous application of salicylic acid and methyljasmonate and the accumulation of caffeine in young leaf tissues and catabolically inactive endosperms

**DOI:** 10.1016/j.dib.2017.05.004

**Published:** 2017-05-10

**Authors:** Avinash Kumar, Gyanendra Kumar Naik, Parvatam Giridhar

**Affiliations:** Department of Plant Cell Biotechnology, CSIR-CFTRI, Mysore 570020, Karnataka, India

## Abstract

Exogenous exposure of coffee plants to 50 μM and 500 μM salicylic acid through liquid hydroponic medium or the exposure to volatile fumes of methyljasmonate was carried out to study the role of salicylic acid and methyljasmonate on the accumulation of caffeine and other methylxanthines like 7-methylxanthine, theobromine and theophylline. Transcript levels of the first, second and third *N*-methyltransferase involved in the core caffeine biosynthetic pathway namely, xanthosine methyltransferase (XMT), methylxanthine methyltransferase (MXMT) and di-methylxanthine methyltransferase (DXMT) was investigated by semi-quantitative RT-PCR for validating the reason behind the changes of caffeine biosynthetic potential under the influence of the two analogues of plant phytohormones. Maturing coffee fruits are known to be biologically inactive with respect to caffeine biosynthetic activity in the endosperms. To understand this, fruits were treated with different doses of salicylic acid in a time-course manner and the de-repression of tissue maturation-mediated knockdown of caffeine biosynthesis by exogenously applied salicylic acid was achieved. In our companion paper [1] it was shown that the repression of NMT genes during the dry weight accumulation phase of maturing endosperm could be relaxed by the exogenous application of salicylic acid and methyljasmonate. A probable model based on the work carried out therein and based on other literature [2], [3], [4] was proposed to describe that the crosstalk between salicylic acid or methyljasmonate and the ABA/ethylene pathway and might involve transcription factors downstream to the signaling cascade.

Specifications Table

**Table:** 

Subject area	Biology.
More specific subject area	Plant Physiology.
Type of data	Graph, Image
How data was acquired	RP-HPLC (LC-10 equipped with SPD10 detector and CR7a recorder, Shimadzu), PCR (Palm Cycler, Corbett Inc.).
Data format	Raw data statistically analyzed.
Experimental factors	Salicylic acid treatment of coffee berries. Salicylic acid and methyljasmonate treatment of coffee seedlings.
Experimental features	One year old coffee seedlings were acclimatized to liquid Hoagland׳s hydroponic medium and the medium was supplemented with 50 and 500 μM salicylic acid or the plants were exposed to 1 μl and 10 μl volatile fumes of methyljasmonate for treatments of 6 h and 12 h. The methylxanthines isolated from young leaves of treated plants was compared to that of untreated plants. After 12 h treatment plants were washed with water and placed back in Hoagland׳s medium for 48 h to serve as control retained sample. The biochemical data was complemented with NMT transcript profiling. Fruits of coffee at the beginning of endosperm dry weight accumulation stage (named as CC5), which is known to have repressed NMT gene expression was harvested and treated with 5, 50, 250 and 500 μM salicylic acid for 6 h, 12 h and 24 h. The methylxanthine contents in the endosperm of the treated fruits was compared to that of untreated fruits and fruits rescued in water control for 48 h after the 24 h salicylic acid treatment.
Data source location	Mysore, Karnataka, India 12.2958°N, 76.6394°E.
Data accessibility	Data is available in this article. Implication of the study accepted in Plant Gene [1].

**Value of the data**

• The data represents methylxanthine metabolite profile and gene expression studies during salicylic acid and methyljasmonate treatment of plants and catabolically active endosperms.
• The data studies the dosage and dosage time on the changes in the metabolite profile of endosperms.
• The data is useful for further studies on the molecular and biochemical regulation of the caffeine metabolism in coffee.

## 1 Data

**Fig. 1:**
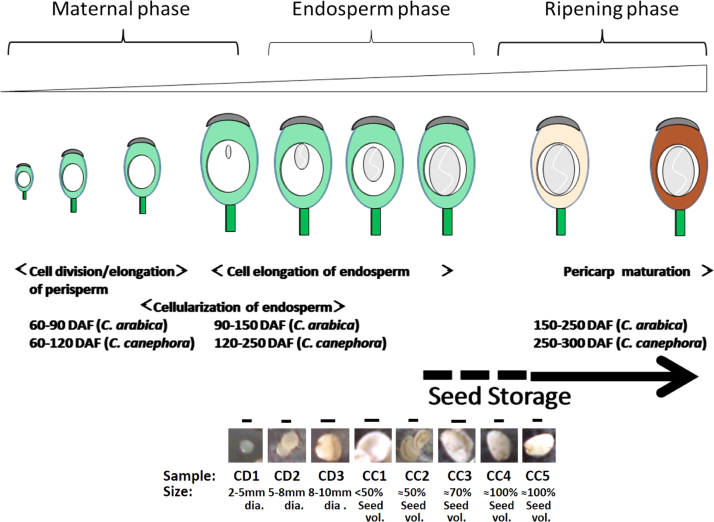
Different phases in the development of fruits in *C. arabica* and *C. canephora*. The major physiological changes during the three phases of development i.e., maternal phase, endosperm phase and ripening phase are common but differ in the time required for developing. The approximate timing with respect to days after flowering (DAF) in *C. canephora* and *C. arabica* is indicated. The lower panel represents the isolated endosperms extracted from fruits from endosperm stage onwards. CYR and CR stages appears like CC5 stages except that the colour of the berry are yellow-red and red, respectively. (Bar=0.5 cm).

**Fig. 2:**
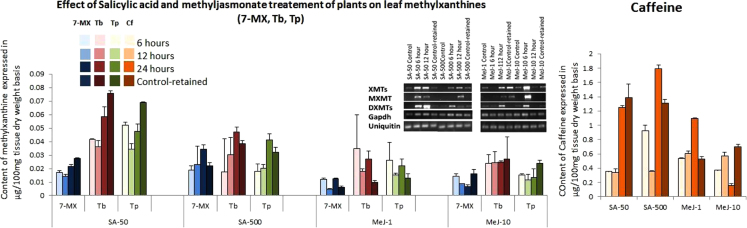
Effects of salicylic acid and methyljasmonate treatments of coffee seedlings on the accumulation of caffeine in young leaves and expression of caffeine biosynthetic NMT transcripts. The changes in the metabolites of caffeine biosynthetic pathway {viz. 7-methylxanthine (7-MX) (blue shades), theobromine (Tb) (magenta shades), and caffeine (Cf) (orange shades)} and the caffeine degradation pathway viz. theophylline (Tp) (green shades). Treatment with salicylic acid at 50 μM (SA-50) and 500 μM (SA-500) and methyljasmonate fumes from 1 μl (MeJ-1) and 10 μl (MeJ-10) lead to enhancement in levels of caffeine. Control-retained refer to plants shifted back to control conditions for 48 h after the 12 h salicylic acid or methyljasmonate treatments. The inlet shows the expression pattern of the three NMTs involved in the caffeine biosynthetic pathway: XMTs (Xanthosine methyltransferase), which convert xanthosine to 7-methylxanthosine and then to 7-methylxanthine (7-MX), MXMTs (Methylxanthine methyltransferase), which convert 7-MX to Tb and DXMTs (7,3 di-Methylxanthine methyltransferases) which convert Tb to Cf. GAPDH and Ubiquitin amplicons were used as the internal reference genes for equal loading of RNA.

**Fig. 3:**
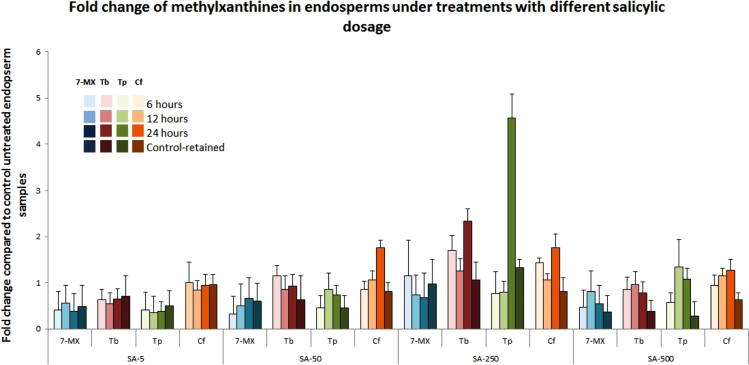
Dosage and time-course experiments for the effect of exogenously applied salicylic acid on de-repression of caffeine biosynthesis in metabolically inactive endosperms (CC5 stage). Fruits from the CC5 stage which show complete repression of the NMTs were challenged with different concentrations of salicylic acid in aqueous solution viz. 5 μM (SA-5), 50 μM (SA-50), 250 μM (SA-250) and 500 μM (SA-500) to observe the augmentation of caffeine by interference of salicylic acid inducible signals with the maturation-triggered repression of caffeine biosynthesis. The treatment was carried out for 6 h, 12 h, and 24 h after which the treated seeds were placed in distilled water for 48 h (Control-retained). The fold changes of 7-MX (different shades of blue), Tb (different shades of magenta), Tp (different shades of green) and Cf (different shades of orange) compared to control untreated seeds are indicated. The error bars represent coefficient of variation of fold change obtained from the analysis of 4–8 seeds.

The data set used in the study involves the experimental results indicating the inducible effects of exogenous exposure of salicylic acid and methyljasmonate on the three NMT transcripts. The data also supplements the study describing the ability of salicylic acid and methyljasmonate in overcoming the maturation-triggered repression of NMT genes during early dry weight accumulation stage [1] of the coffee endosperms (Fig. 1). The involvement of ABA and ethylene pathways during mid to late maturation of fruits (CC5 stage) is anticipated from analysis of the published literature [5], [6]. There occurs a possibility that ethylene [3], [6] and even ABA [4] might correlate or be associated with the down regulation of NMT genes. Moreover, salicylic acid and methyljasmonate are known to cause inducible expression of NMTs in plants other than coffee [2], [3]. Thus, caffeine biosynthesis by virtue of transcriptional regulation of the *N*-methyltransferases involved in the biosynthesis can be regulated by multiple plant hormone pathways like salicylic acid, methyljasmonate, ethylene and possibly even abscisic acid. The study of exogenous stimulation of salicylic acid on maturing endosperm undergoing natural repression of NMTs involved in the core caffeine biosynthetic pathway was carried out in a mid-developmental stage under different dosages and treatment time regimes. The observed changes in the contents of the biosynthetic precursors, 7-methylxanthine, theobromine and caffeine and the degradation product theophylline was interpreted to imply the role of salicylic acid and methyljasmonate (Fig. 2, Fig. 3).

## 2 Experimental design, materials and methods

### 2.1 Treatment of coffee seedlings and coffee fruits

One year old *Coffea canephora* var. Robusta cv. S274 seedlings were acclimatized to liquid Hoagland׳s medium [7] for 48 h in green house. For salicylic acid treatment, 500 mM salicylic acid solution was added to the hydroponic medium to obtain a final concentration 50 μM or at 500 μM. The samples (second leaves from apex) were collected at 6 h and 12 h of treatment. After the 12 h, one set of plants was washed in running water and placed back in control Hoagland׳s medium for the next 48 h to be sampled as the control-retained sample. For methyljasmonate treatments, the Hoagland׳s acclimatized plants were subjected to vapours from either 1 μl or from 10 μl undiluted methyljasmonate in closed plastic chambers. Samples were collected at 6 h and 12 h as before. For methyljasmonate control-retained samples one set of treated seedlings were placed in fresh plastic chambers without the presence of methyljasmonate. Coffee fruits harvested from standing plants were placed in plastic jars containing 5 μM, 50 μM, 250 μM or 500 μM salicylic acid in an aqueous solution. Treatments were carried out for 6 h, 12 h, 24 h and rescued in control Hoagland׳s medium for the next 48 h. The seeds were dissected and the CC5 stage endosperms (Fig. 1) were collected for biochemical analysis. CC5 stage is equivalent to stage E that has shown to be metabolically inactive to caffeine biosynthesis [8].

### 2.2 Methylxanthine isolation and RP-HPLC

Samples were dried at 37 °C, overnight and grinded to fine powder using mortar and pestle. The methylxanthines were extracted in 1 mL of 50% methanol at 70 °C for 10 min as described in [1], [4]. HPLC was carried out using Shimadzu LC 10 liquid chromatograph (Kyoto, Japan) equipped with a dual pump, UV spectrophotometer detector (model SPD 10 A) (270 nM) and the recorder C-R7a Chromatopac using gradient mobile phase as originally described [9]. The biochemical analysis of methylxanthine contents in young leaves of control and treated plants is depicted in Fig. 2 whereas the fold change in different methylxanthines under different dose and time regimes of salicylic acid treatment in the mid-maturation stage (CC5) of endosperm is depicted in Fig. 3.

### 2.3 Statistical validation of biochemical data

The statistical test was performed by calculating the coefficient of variation of the treated and control samples by the formula: $\mathrm{Coefficent}\;\mathrm{of}\;\mathrm{variation}\;\left(\mathrm{CV}\right)=\frac{\mathrm{Standard}\;\mathrm{deviation}}{\mathrm{Mean}}$. The coefficient of variation of fold change was calculated by the formula:

$\mathrm{CV}\;\mathrm{fold}\;\mathrm{change}=\sqrt{({\mathrm{CV Control}}^{2}+{\mathrm{CV Treated}}^{2})}$. The coefficient of variation of fold change was represented in error bars.

### 2.4 RNA isolation and semi-quantitative RT-PCR

RNA was isolated using the Nucleospin II RNA isolation Kit (Machery-Nagel, GmBH and Co., Düren) with on-column DNAase treatment followed by minus-reverse transcriptase-PCR to confirm the absence of genomic DNA contamination in the preparation. ImPromII reverse transcriptase (Promega) was used to prepare the first strand cDNA using 1 µg RNA primed with Oligo-dT_18_ (Qiagen). Diluted cDNA (1:5) was used for semi-quantitative RT-PCR using primers pair for XMTs (NMT123-1F/NMT1-1R), MXMTs or MXMT-like transcript (TSRT-1F/ NMT2-1R or MXMT1-1F/ MXMT1-1R) and DXMTs (CcTS3x-3F/ NMT3-1R) [1], [4]. Glyceraldehyde 3-phosphate dehydrogenase (*gapdh*) and ubiquitin (*ubiquitin*) were used as internal controls. PCR was set in a total reaction of 10 µL using 400 nM primers and 0.5 unit hotstart *Taq* DNA polymerase (Kappa Biosystems). The cycling conditions are mentioned in the companion paper [1] and the PCR products (10 µL) were resolved on 2% agarose gel (w/v) and visualized after ethidium bromide staining in gel documentation system (HeroLab, GmBH Laboratories, Germany). The results of the semi-quantitative RT-PCR is depicted in the inlet picture in Fig. 2.
